# Cost-effectiveness of lung cancer screening and treatment methods: a systematic review of systematic reviews

**DOI:** 10.1186/s12913-017-2374-1

**Published:** 2017-06-19

**Authors:** Farbod Ebadifard Azar, Saber Azami-Aghdash, Fatemeh Pournaghi-Azar, Alireza Mazdaki, Aziz Rezapour, Parvin Ebrahimi, Negar Yousefzadeh

**Affiliations:** 1 Health Promotion Research Center, Iran University of Medical Sciences, Tehran, Iran; 2 Road Traffic Injury Research Center, Tabriz University of Medical Sciences, Tabriz, Iran; 3 Dental and Periodental Research Centre, Tabriz University of Medical Sciences, Tabriz, Iran; 4 Health Management and Economics Research Center, Iran University of Medical Sciences, Tehran, Iran; 5 Department of Health service Management, School of Health Management and Information Sciences & Health Management and Economics Research Center, Iran University of Medical Sciences, Tehran, Iran

**Keywords:** Cost-effectiveness, Lung cancer, Screening, Treatment, Systematic review

## Abstract

**Background:**

Due to extensive literature in the field of lung cancer and their heterogeneous results, the aim of this study was to systematically review of systematic reviews studies which reviewed the cost-effectiveness of various lung cancer screening and treatment methods.

**Methods:**

In this systematic review of systematic reviews study, required data were collected searching the following key words which selected from Mesh: “lung cancer”, “lung oncology”, “lung Carcinoma”, “lung neoplasm”, “lung tumors”, “cost- effectiveness”, “systematic review” and “Meta-analysis”. The following databases were searched: PubMed, Cochrane Library electronic databases, Google Scholar, and Scopus. Two reviewers (RA and A-AS) evaluated the articles according to the checklist of “assessment of multiple systematic reviews” (AMSTAR) tool.

**Results:**

Overall, information of 110 papers was discussed in eight systematic reviews. Authors focused on cost-effectiveness of lung cancer treatments in five systematic reviews. Targeted therapy options (bevacizumab, Erlotinib and Crizotinib) show an acceptable cost-effectiveness. Results of three studies failed to show cost-effectiveness of screening methods. None of the studies had used the meta-analysis method. The Quality of Health Economic Studies (QHES) tool and Drummond checklist were mostly used in assessing the quality of articles. Most perspective was related to the Payer (64 times) and the lowest was related to Social (11times). Most cases referred to Incremental analysis (82%) and also the lowest point of referral was related to Discounting (in 49% of the cases). The average quality score of included studies was calculated 9.2% from 11.

**Conclusions:**

Targeted therapy can be an option for the treatment of lung cancer. Evaluation of the cost-effectiveness of computerized tomographic colonography (CTC) in lung cancer screening is recommended. The perspective of the community should be more taken into consideration in studies of cost-effectiveness. Paying more attention to the topic of Discounting will be necessary in the studies.

## Background

Nowadays cancer is one of the major health problems all over the world [1–4]. The lung cancer is one of the most common cancers worldwide and is the major cause of mortality from cancer in the world [5–8].

In 2008, 1.6 million new cases, and 1.38 million deaths from lung cancer were reported. The highest rates belonged to Europe and North America [9]. Despite the fact that the mortality rate in men has been declining for more than 20 years ago, the mortality rate from lung cancer in women has increased during the past decades, and has stabilized recently [10, 11].

Eastern Europe is accounted for the highest mortality rate from lung cancer in men, while Northern Europe and America have the highest mortality rate among women. In America, black men and women are more affected by the disease [12]. Lung cancer rate is lower in Low and Middle Income Countries (LMICs) [13]. due to increasing in rate of smoking in LMICs, it is expected that the rate increase particularly in China and India in the next few years [14–16].

The most common cause of lung cancer is the prolonged exposure to tobacco smoke, which is the reason of 90% of lung cancers [17–20]. The Percent of lung cancer in people who do not smoke is 15% and the reason is often due to a combination of factors including genetic factors, radon gas, asbestos, and air pollution such as cigarette smoke of another person [21].

Given the general state of health, evaluation of lung cancer may include: lungs photography with X radiation, sputum tests, CT scan, biopsy, breath testing, blood tests, and bone marrow tests [22–28]. The most important treatments for lung cancer include: surgery, laser ablation of malignant lesion, chemotherapy, radiation therapy and photodynamic therapy [29–33].

Currently due to rising costs and limited resources on the one hand and high costs of prevention, screening and treatment of chronic diseases, especially cancer on the other hand, Health care providers are looking for the most effective and cost-effective care. For this reason, economic evaluation studies will be used for this purpose. One of the most important studies in this field is the evaluation of the cost-effectiveness [34]. In this type of analysis, it is necessary to determine the costs and consequences of the use of given technologies, which includes estimates about their value. In this type of analysis, outcomes are measured and expressed through natural units (eg number of life years). By doing this analysis, we can determine that which one of the compared technologies are proper to achieve targets [35, 36].

Fortunately, given the sensitivity and importance of lung cancer, a lot of cost-effectiveness studies conducted by different researchers and valid evidence have been produced in this area. In recent years, due to expansion of these studies in each of these areas of expertise related to lung cancer, some studies have also designed and carried out as a systematic review [37–41].

A systematic review of studies provides integrated and reliable information for users of information [42]. Systematic reviews carried out in different domains with different conclusions can confuse the users of these studies. By considering the expansion of these studies and their scattered results, it is needed to collect and report the results of these types of studies as one cohesive and integrated collection. Therefore, the aim of this study was to systematically review systematic review studies which review the cost-effectiveness in various fields related to lung cancer.

## Methods

This systematic review and Meta-Analysis study was conducted in 2016, using the approach of systematic review adopted from the book entitle “A Systematic Review to Support Evidence-Based Medicine”[43]. Also in accordance with the Preferred Reporting Items for Systematic Reviews and Meta-Analyses (PRISMA) checklist [44–46].

### Eligibility criteria

The inclusion criteria for the study were: systematic review and Meta-analysis studies on the lung cancer patients, studies conducted on Cost- effectiveness, articles published in English language, and articles published from January 2000 to 2march 2016. Excluded criteria from the study included: articles that report other type of economic studies, articles that conduced only in one country, conference presentations, case reports and narrative reviews. Also articles that had low score based on Assessment of Multiple Systematic Reviews (AMSTAR) checklist were excluded.

### Information sources and search strategy

Required data were collected searching the following key words which selected from MeSH: “lung cancer”, “lung oncology”, “lung Carcinoma”, “lung neoplasm”, “lung tumors”, “cost- effectiveness”, “systematic review” and “Meta-analysis”. The following databases were searched: PubMed, Cochrane Library electronic databases, Google Scholar, and Scopus. The complete search strategy for PubMed is shown in Table 1. The search strategy was adapted for each database as necessary. Some of the relevant journals and web sites searched manually. Reference lists of the selected articles also were checked. In the final stage of the literature review we also searched the gray literature and did expert contact.

**Table 1:** Complete search strategy for PubMed

Concept	Search strategy
lung cancer	“lung cancer” OR “lung oncology” OR “lung carcinoma” OR “lung neoplasm” OR “lung tumors”
AND	
Cost- Effectiveness	“cost- effectiveness”
AND	
Systematic review	“Systematic review”, “Meta-analysis”.
**=**	
Completed Search strategy: (((((((“lung cancer”[Title/Abstract]) OR “lung oncology”[Title/Abstract]) OR “lung Carcinoma”[Title/Abstract]) OR “lung neoplasm”[Title/Abstract]) OR “lung tumors”[Title/Abstract]) AND “Cost-Effectiveness”[Title/Abstract]) AND “systematic review”[Title/Abstract]) OR “Meta-analysis” [Title/Abstract]	

### Review process

In the first phase of the review process, an extraction table was designed in which the following items included: first author’s name, study publish year, aim of study, number of all publications included, Meta-Analysis, Time Horizon covered, Quality assessment tool, Screening or treatment, perspective, Discounting, Sensitivity analysis, Incremental analysis and overall result. Validity of the data extraction table was confirmed by experts, and a pilot study was conducted for further improvement of the extraction table. Two authors (RA and A-AS) that had enough experience and knowledge were responsible for independently extraction of the data.

In first phase of article selection, articles with non-relevant titles were excluded. In the second phase, the abstract and the full text of articles were reviewed to include those articles matching the inclusion criteria. Reference management (Endnote X5, Thomson Reuters, and Philadelphia, PA 19130, USA) software was used for organizing and assessing the titles and abstracts, as well as for identifying the duplicate entries.

### Quality assessment

Two reviewers (RA and A-AS) evaluated the articles according to the checklist of “assessment of multiple systematic reviews” (AMSTAR) tool [47]. Responses of the AMSTAR tool are ‘Yes’, ‘No’, ‘Can’t Answer’, or ‘Not Applicable’, with yes being rated as ‘1’, and ‘no’, ‘can’t answer’, or ‘not applicable’ rated as ‘0’. Based on this tool, reviews were rated as ‘low’ from 1 to 4, ‘moderate’ from 5 to 7 or ‘high’ from 8 to 11 quality. Articles with “low” quality were excluded. Controversy cases between reviewers were referred to a third author (AFA-F).

### Data analysis

The retrieved data were briefed in extraction table and finally, for mapping and categorizing the result a manual Content-Analysis was used. This is a method to detecting, categorizing and reporting themes from text and is very useful in analyzing qualitative data [48–51].

## Results

**Fig. 1:**
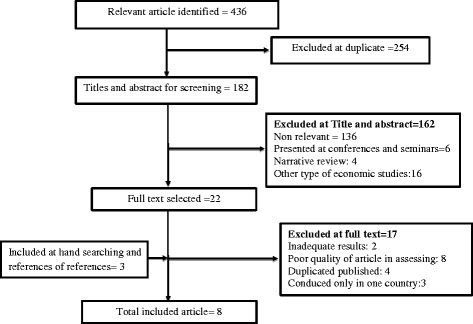
Literature review and retrieval flow diagram

In this study, out of 436 articles, finally 8 articles were completely related to the study objective included in the analysis (Fig 1).

As seen in Fig. 1, 254 articles have been removed due to duplication between databases, 162 articles have been omitted by reviewing their abstracts and 17 papers were excluded by reviewing their full texts. Also In assessing the quality of articles, 8 articles was deleted.

The results of extracted data from entered articles are summarized in Table 2.

**Table 2:** Characteristics of the included studies

Reference	Aim of study	No. of all publications included	Meta-Analysis	Time Horizon covered	Quality assessment tool	Screening or treatment
Clegg, Scott et al. 2001 [84]	Examines the cost-effectiveness of four of the newer drugs –vinorelbine, gemcitabine, paclitaxel and docetaxel used for treating the most common type of lung cancer (non-small-cell lung cancer).	16	NO	1995–2000	appended appraisal questions	treatment
Lange, Prenzler et al. 2014 [85]	review and assess the economic evidence of treatments with targeted agents in advanced: Non-small cell lung cancer	19	NO	2000–2013	The Quality of Health Economic Studies (QHES)	treatment
Bongers, Coupe et al. 2012 [86]	comparing the new agents docetaxel, paclitaxel, vinorelbine, gemcitabine and pemetrexed, and the targeted therapies erlotinib and gefitinib with one another	10	NO	2001–2010	British Medical Journal (BMJ) 35-item checklist	treatment
Raymakers, Mayo et al. 2016 [87]	cost-effectiveness of lung cancer screening using low-dose computed tomography (LDCT)	13	NO	2000–2014	Drummond checklist	Screening
Maher, Miake-Lye et al. 2012 [88]	cost and cost-effectiveness of the different approaches in Treatment of Metastatic Non-Small Cell Lung Cancer	22	NO	1996–2010	-	treatment
Brown, Pilkington et al. 2013[89]	Cost-effectiveness of first-line chemotherapy for patients with advanced and/or metastatic NSCLC.	6	NO	1980–2010	35-item list described by Drummond and Jefferson	treatment
Cao, Rodrigues et al. 2012[90]	describing cost-effectiveness of positron-emission tomography(PET) in staging of non–small-cell lung cancer (NSCLC) and management of solitary pulmonary nodules (SPN)	18	NO	1950–2010	Quality of Health Economic Studies (QHES)	Screening
Black, Bagust et al. 2006 [58]	examine the cost-effectiveness of screening for lung cancer using computed tomography (CT)	6	NO	1994–2005	checklist developed by Drummond and colleagues	Screening

In the process of investigating the 8 systematic reviews, information of 110 articles were discussed in total. None of the reviewed studies had used the meta-analysis method. The desired time frame of reviewed papers varied between from 1950 to 2014. The Quality of Health Economic Studies tool (QHES) and Drummond checklist were mostly used in assessing the quality of articles. Authors of the 5 articles from 8 reviewed papers had focused on the cost-effectiveness of lung cancer treatments and authors in 3 other articles were assessing the cost-effectiveness of lung cancer screening methods.

Exclusive information related to the cost-effectiveness (perspective, Discounting, Sensitivity analysis, Incremental analysis and overall result) has been presented in Table 3.

**Table 3:** Characteristics of the included studies

Reference	perspective	Discounting	Sensitivity analysis	Incremental analysis	Overall result
Clegg, Scott et al. 2001 [84]	Social:3 Health:6 Payer:7 Not reported: 0	16	16	16	Vinorelbine has been reported to deliver cost savings or low incremental cost compared with best supportive care. Gemcitabine and paclitaxel have also led to small but acceptable incremental costs over BSC.
Lange, Prenzler et al. 2014 [85]	Social:1 Health:7 Payer:11 Not reported: 0	17	12	17	First-line maintenance treatment with erlotinib compared to Best Supportive Care (BSC) can be considered cost-effective. In comparison to docetaxel, erlotinib is likely to be cost-effective in subsequent treatment regimens as well. The insights for bevacizumab are miscellaneous. There are findings that gefitinib is cost-effective in first- and second-line treatment
Bongers, Coupe et al. 2012 [86]	Social: 0 Health:8 Payer:2 Not reported: 0	3	NS	8	In first-line treatment, gemcitabine + cisplatin was cost effective compared with other platinum-based regimens (paclitaxel, docetaxel and vinorelbine). In second-line treatment, docetaxel was cost effective compared with best supportive care; erlotinib was cost effective compared with placebo; and docetaxel and pemetrexed were dominated by erlotinib.
Raymakers, Mayo et al. 2016 [87]	Social: 4 Health:1 Payer:5 Not reported:3	8	12	12	Results ranged from US$18,452 to US$66,480 per LYG and US$27,756 to US$243,077 per QALY gained for repeated screening. The cost-effectiveness of a lung cancer screening program using LDCT remains to be conclusively resolved. It is expected that its cost-effectiveness will largely depend on identifying an appropriate group of high risk subjects
Maher, Miake-Lye et al. 2012 [88]	Social: 1 Health:4 Payer:17 Not reported:0	NS	NS	NS	There are a large number of published cost-effectiveness analyses, but approximately two-thirds of such studies are supported by the makers of the drugs being assessed. Invariably, studies supported by the makers concluded that their drug was cost-effective. Of the cost-effectiveness analyses not supported by industry, the addition of bevacizumab to first-line therapy was found in one study to be not cost-effective, erlotinib was found in one study to be marginally cost-effective, and the differences between erlotinib and docetaxel maintenance therapy were slight in another study (GRADE = low).
Brown, Pilkington et al. 2013[89]	Social: 1 Health:2 Payer:3 Not reported: 0	0	6	6	It is clear from the preceding sections that, although there exists published cost-effectiveness evidence comparing different first-line chemotherapy regimens for patients with NSCLC, very few studies are directly helpful to decision-makers, because the studies not estimate ICERs in terms of cost per QALY gained
Cao, Rodrigues et al. 2012 [90]	Social: 0 Health:0 Payer:18 Not reported: 0	NS	18	18	The mean cost of PET was $1478. The cost-effectiveness metrics used in these studies were variable depending on sensitivity and specificity of diagnostic tests used in the models, probability of malignancy, and baseline strategy.
Black, Bagust et al. 2006 [58]	Social: 1 Health:1 Payer:1 Not reported: 3	5	5	5	The magnitude of cost-effectiveness ratios reported very widely. All six made the fundamental assumption that screening with CT for lung cancer reduced mortality. At the current time, there is no evidence to support that assumption.

*NS* not specified clearly

**Fig. 2:**
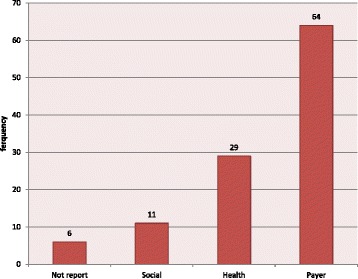
Frequency distributions of referenced perspectives in the studies (total = 110)

In this study, mentioned perspectives in the studies were classified into four groups: Social, Health, Payer and not reported. Results of repetition of each of the four groups are shown in Fig. 2. As seen in Fig. 2, most perspective was related to the Payer and the lowest belonged to Social. In six studies, the perspective was not reported.

**Fig. 3:**
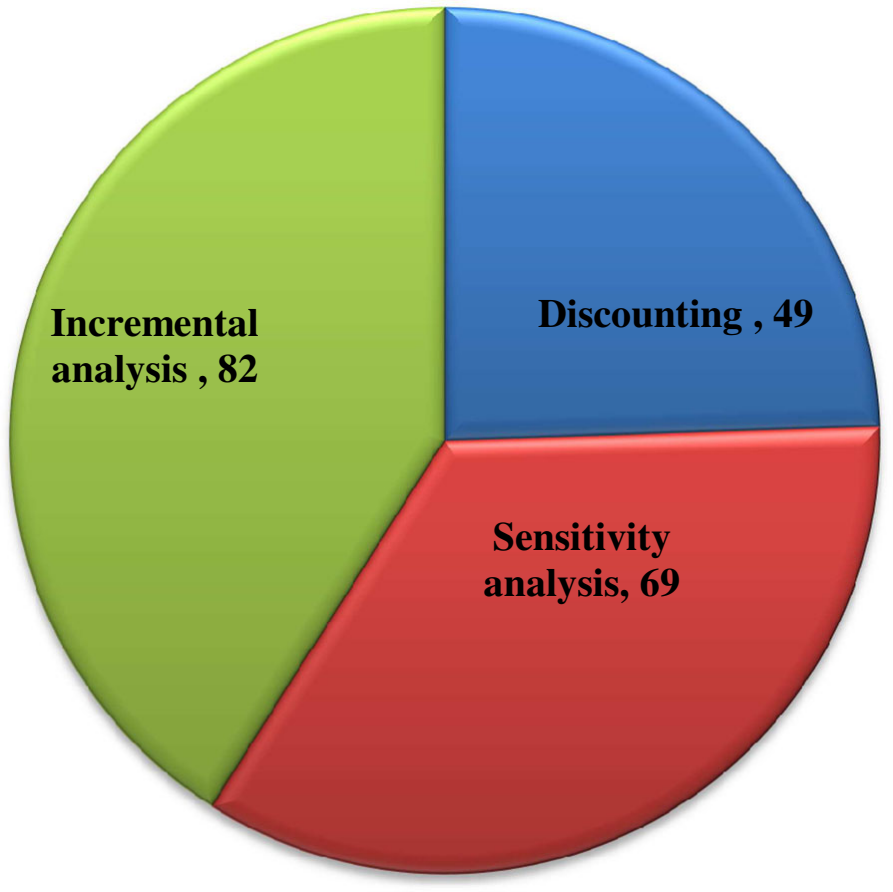
Frequency distribution of cases referred to the Discounting, Sensitivity analysis and Incremental analysis in110 articles among 8 systematic reviews embodied in the study

Among 8 reviewed studies, Discounting and Sensitivity analysis weren’t mentioned in two articles and Incremental analysis wasn’t referred to in one study. The frequency distribution of cases referred to the Discounting, Sensitivity analysis and Incremental analysis in110 articles among 8 systematic reviews which were compiled in this study, is shown in Fig. 3. As seen in Fig. 3, most of cases refer to Incremental analysis (in 82% of cases) and the lowest rate of reference belonged to Discounting as well (in 49% of cases).

Results of the Quality Assessment of the entered articles to the study are shown in Table 4. All of the 8 articles which were embodied in the study had high quality and all cases had been regarded in 2 articles (score of 11). Also the average quality score of the articles were evaluated to be high (9.25 out of 11).

**Table 4:** AMSTAR (assessment of multiple systematic reviews) checklist

Reference	1	2	3	4	5	6	7	8	9	10	11	Score from11
1. Clegg, Scott et al. 2001 [84]	Y	Y	Y	Y	Y	Y	Y	Y	Y	Y	Y	11
2. Lange, Prenzler et al. 2014 [85]	N	Y	Y	Y	N	Y	Y	Y	Y	Y	Y	9
3. Bongers, Coupe et al. 2012 [86]	N	Y	Y	Y	N	Y	N	Y	Y	Y	Y	8
4. Raymakers, Mayo et al. 2016 [87]	Y	Y	Y	Y	Y	Y	N	Y	N	N	Y	8
5. Maher, Miake-Lye et al. 2012 [88]	Y	Y	Y	Y	Y	Y	N	N	Y	Y	Y	9
6. Brown, Pilkington et al. 2013[89]	Y	Y	Y	Y	Y	Y	Y	Y	N	Y	Y	10
7. Cao, Rodrigues et al. 2012 [90]	N	N	Y	Y	Y	Y	Y	Y	Y	N	Y	8
8. Black, Bagust et al. 2006 [58]	Y	Y	Y	Y	Y	Y	Y	Y	Y	Y	Y	11

1- Was an “a priori” design provided?, 2- was there duplicate study selection and data extraction?, 3- was a comprehensive literature search performed?, 4- was the status of publication (ie, grey literature) used as an inclusion criterion?, 5- was a list of studies (included and excluded) provided?, 6- were the characteristics of the included studies provided?, 7- Was the scientific quality of the included studies assessed and documented?, 8-Was the scientific quality of the included studies used appropriately in formulating conclusions?, 9- Were the methods used to combine the findings of studies appropriate?, 10- was the likelihood of publication bias assessed?, 11- Was the conflict of interest included?

*Y* yes, *N* no, *CA* can’t answer, *NA* not applicable

## Discussion

Results of the present study indicated that in the eight reviewed systematic reviews, information of 110 papers was summarized. None of the reviewed studies had used the meta-analysis method. The QHES and Drummond checklist were mostly used in assessing the quality of articles. Authors of the 5 articles from 8 reviewed articles had focused on the cost-effectiveness of lung cancer treatments and goal of the researchers in 3 other articles were assessing the cost-effectiveness of methods for screening lung cancer. The most perspective referred to Payer (64 times) and the lowest was related to Social (11 times). Six studies not report the perspective of study. Most cases referred to Incremental analysis (82%) and the lowest rate of reference belonged to Discounting (in 49% of cases). The average quality score of included systematic reviews was calculated 9.2% from 11.

One of the major treatments that were mentioned in numerous studies is the targeted drug therapy. Targeted therapies are newer treatments that work by targeting specific abnormalities in cancer cells. Targeted Therapeutic options for Lung Cancer Treatment include: bevacizumab, Erlotinib and Crizotinib that the cost-effectiveness of this type of therapies in the treatment of lung cancer has been demonstrated in many studies [52–57]. Thus, this therapy can be one of the options for decision makers and politicians to choose and use in the clinical settings of their own countries. Although the assessment of the cost-effectiveness of this therapeutic method in the local environment of each country should not be forgotten before choosing this treatment; since the cost-effectiveness of procedures and treatments can vary from one environment to another. Also the results of these kind of studies can be tarnished by many factors such as pharmacy company sponsorship for those undertaking the economic evaluations.

Results of a systematic review by Black, Bagust et al. (2006) [58] failed to demonstrate the cost-effectiveness of computed tomography (CT) in lung cancer screening. However results of a study by Hanly, Skally et al. (2012) [59] and Kriza, Emmert et al. (2014) [60], meanwhile, indicated the cost-effectiveness of this approach in patients with colon cancer. The probable reason for this difference could be the type of computed tomography in these studies, because the computerized tomographic colonography (CTC) had been used in the both studies on colon cancer patients. Thus, it appears that CTC is more cost-effective compared to CT. However, this should be investigated and evaluated in patients with lung cancer.

Although systematic reviews of studies have their own value, their value is appreciably enhanced when combined with meta-analysis methods [61–63]. Two possible reasons for not using meta-analysis are discussed in the literature. First scholars do not know how to do it, in which case providing the necessary training on how to perform meta-analysis seems quite necessary. Secondly, methodological and data problems in the process of study do not allow the researchers to carry out the meta-analysis method. To address this problem use of specific guidelines for publication as well as training of scholars about the methods of carrying out a high quality research can have considerable results.

According to the study results, among various assessment tools, QHES and Drummond checklist were mostly used in assessing the quality of articles. QHES designed by Chiou and colleagues in 2003 in America and consists of 16 questions [64]. Drummond checklist contains 35 questions in three sections: Study design (7 questions), Data collection (14 questions) and Analysis and interpretation of results (14 questions) [65]. Due to the comprehensiveness and applicability of these tools in assessing the quality of economic evaluation studies, Psychometric of these tools and using them in different countries are recommended.

Based on results of the study, among existing perspectives, perspective of community had the lowest references in the studies. In addition, it should be noted that cancer imposes considerable cost to the health care system and third-party payers. As a matter of fact, cancer imposes a lot of direct and indirect financial and psychological costs to community and families [66–71]. According to findings of a study by Bradley et al. (2008) in United States, mortality due to lung cancer accounted about 27% of productivity costs [72]. One of the possible reasons for comparatively little attention of researchers to the perspective of community is difficulties in calculating costs in this method. The possible reason for these difficulties and problems can be mainly derived from the unavailability of data and the difficulties of collecting data in this area compared with the perspective of health system and third-party payers [73–75]. Hence, designing a community-based information system for efficiently and effectively collection of the community-level information seems necessary.

Between Discounting, Sensitivity analysis and Incremental analysis, the lowest rate of reference was respectively estimated to be for Discounting (43%). Given the importance of this topic in economic evaluations, failure in attention to this issue can distort the results of economic evaluations and reduce the applicability of the results [76–81].

Discounting rate was also reported lower than the Sensitivity analysis and Incremental analysis, in a systematic review by Leung, Chan et al. (2013) [82] which evaluated the cost-effectiveness of pharmaceutical therapies for metastatic colorectal cancer (MCRC). Discounting is a method to estimate the present value or the current value of cash flows, which are available at a specific time sequence in the future [83]. One of the reasons for paying less attention to the discounting may be its nature, because “future time” is the main factor in the calculation of this index. Also due to considerable uncertainty about the future in the healthcare system compared with the other sectors, the calculation of this index is difficult and unreliable. Another possible reason that can be cited in this context is that despite the interest of scientists in this issue, they may neglect in reporting this index in the article or the scholars who conducted systematic reviews may be wrong in the process of extracting information.

. In current study due to existing defriends in report of studies results and some methodological issue, we cannot conduct Meta-Analysis. Errors may be occurred in the extraction and analysis of the results of this systematic review as it is possible in the other studies. It is noteworthy that most efforts have been made to apply highest possible accuracy in extracting and analyzing data in this study.

## Conclusion

The results showed that the targeted therapy options (bevacizumab, Erlotinib and Crizotinib) can be an option for treatment of lung cancer. Evaluation of the cost-effectiveness of the computerized tomographic colonography (CTC) in lung cancer screening is recommended. Use of meta-analysis techniques is required in this field. Psychometric of Drummond checklist and The QHES is recommended in different countries. Perspective of community should be taken into consideration in cost-effectiveness evaluation studies. Paying more attention to the topic of Discounting will be necessary in future researches.
